# Valproic Acid as an Adjuvant Treatment for Generalized Convulsive Status Epilepticus in Adults Admitted to Intensive Care Units: Protocol for a Double-Blind, Multicenter Randomized Controlled Trial

**DOI:** 10.2196/22511

**Published:** 2021-02-24

**Authors:** Tarek Sharshar, Omar Ben Hadj Salem, Raphaël Porcher, Lamiae Grimaldi-Bensouda, Nicholas Heming, Bernard Clair, Eric Azabou, Aurélien Mazeraud, Benjamin Rohaut, Hervé Outin

**Affiliations:** 1 Groupement Hospitalo-Universitaire Paris Psychiatrie et Neurosciences Paris France; 2 Université de Paris Paris France; 3 Centre Hospitalier Poissy Saint Germain en Laye Poissy France; 4 Center for Clinical Epidemiology Assistance Publique Hôpitaux de Paris Hôtel Dieu Hospital Paris France; 5 Clinical Research Unit Ambroise Paré Hospital University of Versailles Saint-Quentin en Yvelines Saint-Quentin en Yveline France; 6 Centre Hospitalo-Universitaire Raymond Poincaré Assistance de Paris - Hôpitaux de Paris Garches France; 7 Centre Hospitalo-Universitaire Pitié Salpétrière Paris France; 8 Sorbonne Université Paris France

**Keywords:** generalized convulsive status epilepticus, intensive care unit, seizure, valproic acid

## Abstract

**Background:**

Generalized convulsive status epilepticus (GCSE) is a frequent medical emergency. GCSE treatment focuses on the administration of benzodiazepines followed by a second-line antiepileptic drug (AED). Despite this stepwise strategy, GCSE is not controlled in one-quarter of patients and is associated with protracted hospitalization, high mortality, and long-term disability. Valproic acid (VPA) is an AED with good tolerability and neuroprotective properties.

**Objective:**

This study aims to demonstrate that administration of VPA as an adjuvant for first- and second-line treatment in GCSE can improve outcomes.

**Methods:**

A multicenter, double-blind, randomized controlled trial was conducted, comparing VPA with a placebo in adults admitted to intensive care units (ICUs) for GCSE in France. GCSE was diagnosed by specifically trained ICU physicians according to standard criteria. All patients received standard of care, including a benzodiazepine and a second-line AED (not VPA), at the discretion of the treating medical team. In the intervention arm, VPA was administered intravenously at a loading dose of 30 mg/kg over 15 minutes, followed by a continuous infusion of 1 mg/kg/hour over the next 12 hours. In the placebo group, an identical intravenous administration of 0.9% saline was used. The primary outcome was the proportion of patients discharged alive from the hospital by day 15. Secondary outcomes were frequency of refractory and super refractory GCSE, ICU-related morbidity, adverse events related to VPA, and cognitive dysfunction at 3 months. Statistical analyses will be performed according to the intent-to-treat principle.

**Results:**

The first patient was randomized on February 18, 2013, and the last patient was randomized on July 7, 2018. Of 248 planned patients, 98.7% (245/248) were enrolled across 20 ICUs. At present, data management is still ongoing, and all parties involved in the trial remain blinded.

**Conclusions:**

The Valproic Acid as an Adjuvant Treatment for Generalized Convulsive Status Epilepticus (VALSE) trial will evaluate whether the use of VPA as an adjuvant for first- and second-line treatment in GCSE improves outcomes.

**Trial Registration:**

ClinicalTrials.gov NCT01791868; https://clinicaltrials.gov/ct2/show/NCT01791868.

**International Registered Report Identifier (IRRID):**

DERR1-10.2196/22511

## Introduction

### Background and Rationale

Generalized convulsive status epilepticus (GCSE) is a diagnostic and therapeutic emergency. Mortality or long-term neurological deterioration increases with time to successful seizure termination [1]. In-hospital mortality reaches 20% to 40% in refractory GCSE, and 23% of patients with GCSE have permanent disability [2]. Underlying etiology, older age, and duration of seizure are the main predictors of unfavorable outcomes [2]. Guidelines have been established to improve the detection, management, and outcome of GCSE [3]. GCSE is defined as a convulsive seizure lasting more than 5 minutes or as consecutive seizures without recovery of consciousness between seizures [4]. Stepwise antiepileptic therapy is recommended. Emergent initial therapy consists of the administration of benzodiazepine (ie, lorazepam, clonazepam, diazepam, or midazolam). If GCSE is not controlled, current guidelines recommend second-line antiepileptic drugs (AEDs), including phenytoin or fosphenytoin, valproic acid (VPA), phenobarbital, or levetiracetam, administered intravenously [3]. Despite the proven efficiency of this stepwise strategy, cessation of seizures is still not obtained in about a quarter of patients [5,6], and GCSE remains associated with prolonged hospitalization and long-term disability. To improve seizure control and long-term outcomes, randomized clinical trials (RCTs) were therefore undertaken to determine the most efficient second-line AEDs [5-8].

Another approach consists of combining first- and second-line AED therapy with adjuvant treatment [5]. The addition of hypothermia did not improve neurological outcome in GCSE [9]. We reasoned that a strategy based on the administration at the time of intensive care unit (ICU) admission of a treatment exhibiting antiepileptic and neuroprotective properties as well as being well tolerated (ie, not inducing or requiring sedation) might be beneficial. At the time of the design of our RCT (ie, 2012), VPA seemed to be one of the best options [10]. Indeed, at that time, French guidelines did not recommend VPA as a second-line AED, except for GCSE, obviously related to its withdrawal [11]. Until recently, only six randomized trials compared VPA with either phenytoin, phenobarbital, or diazepam [12]. Their meta-analysis indicated a similar rate (77%) of seizure cessation with VPA compared with other AEDS [13]. However, these studies had major limitations, including single-center designs or their small size [13-15]. Interestingly, a very recent multicenter trial *Established Status Epilepticus Treatment Trial* (ESETT) showed on a large cohort that VPA was equivalent to fosphenytoin and levetiracetam as second-line AEDs in adult patients for the early control of seizures [16]. It must be noted that VPA is only prescribed during GCSE by 16% of neurologists, mostly when GCSE is refractory [17]. A French survey reported that VPA was used as a second-line AED in approximately 9% of cases [18]. Therefore, we think that addressing the effect of VPA as a complementary treatment to the recommended stepwise antiepileptic strategy remains relevant, notably because of its antiepileptic efficacy [14,15], potential neuroprotective effect [10], good tolerance, and compatibility with other AEDs [19].

We hypothesized that 1 mg/kg over 12 hours of VPA intravenously after a loading dose of 30 mg/kg over 15 minutes in patients admitted to the ICU for GCSE, in addition to the recommended stepwise antiepileptic strategy would increase the number of patients discharged alive from the hospital by day 15 after GCSE onset.

### Objectives

#### Primary Objective

The primary objective is to assess whether VPA increases the proportion of patients with GCSE discharged alive from the hospital on day 15 following ICU admission after adjustment for age and existence of primary brain insult. The effectiveness of AED in GCSE is commonly assessed in terms of their ability to rapidly control seizures. However, measuring the effect of VPA on a longer-term outcome such as hospital discharge is clinically relevant and easily assessable. Indeed, prompt hospital discharge indicates that the global management of GCSE was appropriate with early control of epilepsy, prompt treatment of the underlying cause, low mortality, and a short hospital stay. Furthermore, this end point has previously been reported, facilitating sample size calculation. [9]. In the Veterans Administrations Cooperative study comparing different first-line treatments of status epilepticus, only 50% of patients enrolled in the placebo arm had been discharged from the hospital on day 30 [8].

#### Secondary Objective

The secondary objective is to determine whether VPA decreases the rates of seizures, refractory and superrefractory GCSE, ICU-related morbidity, and poor neurological outcome at 3 months. We will control for the underlying cause of GCSE and monitor the side effects of VPA.

### Trial Design

**Figure 1:**
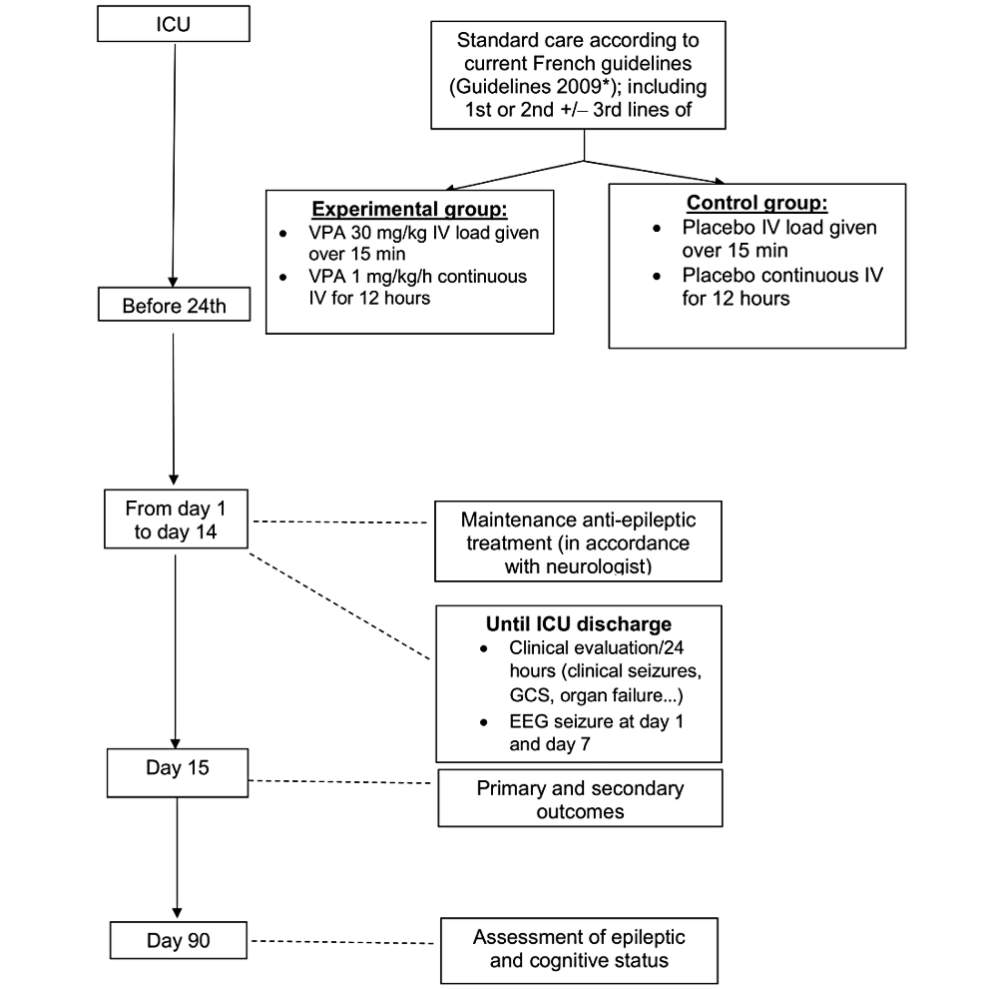
Study design. AED: antiepileptic drug; EEG: electroencephalogram; GCS: Glasgow Coma Scale; ICU: intensive care unit; IV: intravenous. *Guidelines from the French Intensive Care Society [11].

The Valproic Acid as an Adjuvant Treatment for Generalized Convulsive Status Epilepticus (VALSE) trial is a multicenter, parallel-group, double-blind RCT comparing the adjunction of intravenous VPA against placebo in patients admitted to the ICU for GCSE, in addition to firstand second-line AEDs and standard ICU care (Figure 1).

## Methods

### Study Setting

A total of 20 centers, including 10 general hospitals and 10 university hospitals, participated in this study. Factors determining which centers were selected to participate were capacity to include patients, knowledge, and adherence to current guidelines on the management of GCSE. Several participating physicians were involved in drafting the recommendations for management of GCSE issued by the French Intensive Care Society [11]. All participating centers had previously participated in clinical trials. Finally, training on the study procedures was provided to all participating staff members. Documents required for the study, including the study protocol and management guidelines, were available in each participating ICU.

### Eligibility Criteria

Adult patients were eligible if admitted to the ICU for GCSE, defined as 5 minutes or more of continuous generalized clinical seizure activity or recurrent generalized seizure without recovery of consciousness between seizures [11] provided that antiepileptic treatment had been initiated before inclusion either within 6 hours if GCSE was controlled by the time of inclusion (ie, absence of seizure irrespective of the level of consciousness) or within 24 hours if GCSE persisted or reoccurred. The former criteria aimed to include patients during the early stage of GCSE; the latter subcriteria aimed at enrolling patients with superrefractory GCSE, as we reasoned that VPA could be beneficial for both types of population. The reported proportion of patients with superrefractory GCSE is approximately 15% [20]. The patient’ s informed consent or next of kin assent was obtained before inclusion. Alternately, deferred patient consent was obtained.

The exclusion criteria were as follows: (1) nonconvulsive status epilepticus; (2) postanoxic status epilepticus; (3) the primary clinical team decided to treat the patient with VPA before randomization; (4) GCSE occurred during hospitalization for a disease with an expected length of stay >15 days; (5) expected ICU length of stay <12 hours; (6) life expectancy <3 months; (7) women of childbearing age (>17 and <50 years), pregnant women, or women with eclampsia; (8) VPA contraindications, including liver disease (preexisting chronic or acute hepatitis, cirrhosis, hepatitis B or C virus or family history of acute hepatitis), porphyria, hypersensitivity to VPA or derivatives, treatment with mefloquine or hypericum-containing drugs; (9) included in any treatment trial; (10) previously been included in this trial; and (11) no health insurance coverage; and (12) being under guardianship. To reduce recruitment bias, we did not discourage the primary medical or ICU team to use VPA as a secondary AED.

All patients admitted for GCSE in one of the participating ICUs were screened for eligibility by the ICU physicians round the clock and reasons for nonrandomization were collected. In each participating center, GCSE diagnosis was made by ICU physicians specifically trained for.

### Who Will Take Informed Consent?

Written informed consent of the patient had to be obtained by the investigator of the participating center. In case of impaired consciousness, the investigator sought written consent from the next of kin. If the latter was not present, the patient could be included, as deferred consent was approved by the Ethics Committee, according to the French law (Art L1122-1-2 du Code de la Santé Publique). As soon as the patient’s status allowed, written informed consent for the continuation of the research and analyses of the data had to be obtained. A copy of the consent form was given to every patient. The investigator had to keep the original copy in his archives for a minimum of 15 years. A third copy was archived by the promoter.

### Interventions

#### Explanation for the Choice of Comparators

As VPA was tested as an adjuvant therapy, patients were treated with AED according to local guidelines [11], which recommended clonazepam (or diazepam) as a first-line AED and phenobarbital or fosphenytoin as second-line drugs. Therefore, belonging to the comparator or the experimental group did not change the recommended treatment administration. Saline 0.9% administration was used as a placebo to control the intervention.

#### Intervention Description

In the intervention group, VPA treatment consisted of intravenous administration of a loading dose of 30 mg/kg over 15 minutes followed by a continuous intravenous dose of 1 mg/kg/hour over the next 12 hours. Given that the half-life of VPA is approximately 16 hours, this protocol aims to rapidly reach and maintain therapeutic plasma levels of VPA. In the control group, an identical intravenous administration of 0.9% saline as a bolus and continuous infusion was used as a placebo for VPA.

#### Criteria for Discontinuing or Modifying Allocated Interventions

A centralized phone and email center answered participating centers’ questions regarding patient eligibility or management and declaration of any adverse event during the trial period. Reasons for any experimental treatment being discontinued before full-dose administration were recorded.

#### Strategies to Improve Adherence to Interventions

The participating teams were informed of the course of the study and reminded of the main elements of the trial on a monthly basis. Blood samples for VPA dosage were drawn before (T0), 15 minutes, and 12 hours after VPA administration to assess whether VPA has been appropriately administered in the intervention group and not mistakenly administered in the control group.

### Relevant Concomitant Care Permitted or Prohibited During the Trial

In both groups, patients benefited from standardized care, including antiepileptic therapy, control of secondary brain injuries, etiological investigations, and neurological monitoring. As VPA was tested as an adjuvant therapy, patients were treated with AED according to local guidelines [11], which recommended clonazepam (or diazepam) as a first-line AED and phenobarbital or fosphenytoin as second-line drugs. Recommended anticonvulsant therapy for refractory and superrefractory GCSE includes the infusion of sedative agents (ie, propofol or midazolam) and thiopental, respectively [11]. Maintenance antiepileptic treatment was started between the 12th and 24th hours and was decided by the local physician independent of the trial protocol. In patients with a history of epilepsy, recommendations dictated the resumption of antiepileptic medications that controlled seizures in the patient before GCSE onset.

Prevention of secondary brain injuries was based on temperature, mean blood pressure, blood glucose, sodium levels, PaO_2_, and PaCO_2_ control. Etiological investigations were conducted by the physicians in charge of the patient. Patients were assessed neurologically every 4 hours using the Glasgow Coma Scale (GCS) or the Richmond Assessment Sedation Scale. When the GCS was ≤8, pupillary light reflex, and corneal and cough reflexes were assessed. In the absence of coma or sedation, delirium was detected using the Confusion Assessment Method in the ICU every 12 hours. The presence of focal neurologic signs and abnormal movements were systematically collected every 4 hours. This standardized neurological examination helped assess the duration of seizures, relapse of GCSE, progression to refractory and superrefractory GCSE, neurological deterioration, delay for arousal, and post-GCSE delirium. These standardized regular and frequent neurological assessments are used to potentially trigger complementary investigations such as imaging or electrophysiological tests. In every patient, at least one 30-minute electroencephalogram (EEG) was performed within 24 hours of admission and another one between days 2 and 7. EEGs were interpreted by the referent neurophysiological team of the participating center. All EEGs were stored to be sent for a posteriori adjudication by a group of experts blinded to the randomization arm.

In both intervention groups, serum samples were obtained before and 15 minutes and 12 hours after the administration of the VPA load to measure serum VPA concentrations. Samples were stored at −20°C in the participating centers before being sent to the Department of Pharmacology and Toxicology of the Raymond Poincaré Teaching Hospital (Garches, France) for centralized VPA measurements.

### Provisions for Posttrial Care

In France, research promoter insurance offers a subsequent period of 10 years from the end of the research. Consequently, in the event of poststudy damage to a subject related to their participation in research, the complaint would be admissible as soon as it occurs during this period.

### Outcomes

The *primary outcome* was the proportion of patients discharged alive from hospital to their home or to a long-term care facility on day 15. Therefore, death within the first 15 days or a medical reason to keep the patient hospitalized beyond day 15 was considered a poor outcome. Conversely, hospitalization lasting more than 15 days was not considered a failure if the patient was declared fit for discharge from hospital but remained hospitalized because of social issues or a lack of bed availability in recovery facilities. The primary end point (ie, hospital status at day 15) will be collected by a blinded investigator.

*Secondary outcomes* were (1) frequency of refractory and superrefractory GCSE [20], (2) morbidity related to the ICU stay, (3) rates and types of VPA adverse effects, and (4) cognitive dysfunction at 3 months.

We aimed to determine whether intravenous VPA as an adjuvant AED prevented the recurrence of refractory GCSE within 3 months, reduced ICU-related complications, and improved cognitive status at 3 months, irrespective of the cause of GCSE.

### Participant Timeline

For any patient admitted for GCSE into one of the participating ICUs, investigators checked the patient’s eligibility criteria for the VALSE study. If a patient was eligible but unable to provide free and informed consent, the investigator attempted to obtain signed consent from the patient’s next of kin or included the patient according to the deferred consent procedure. The investigator notified the patient or his next of kin of enrollment as early as possible and sought the patient’s consent to continue whenever possible. The patient was then randomized through a centralized, secured website to receive either placebo or VPA.

Within 24 hours of inclusion, a 30-minute standard EEG was performed. Antiepileptic relay therapy was started 12 hours after completion of the treatment infusion or later in case of progression to refractory GCSE. Blood samples for VPA dosage were drawn before (T0), 15 minutes, and 12 hours after VPA administration (Table 1).

**Table 1:** Trial visits summary.

Time point		Enrollment	Allocation	Postallocation			Close-out				
		−*t*_1_	0	*t0*	*15 min*	*12 h*	Day 1	Days 2,3,4,7	Day 15	Days 15-90	Day 90
**Enrollment**											
	Eligibility screen	✓^a^	—^b^	—	—	—	—	—	—	—	—
	Informed consent	✓	—	—	—	—	—	—	—	—	—
	Allocation	—	✓	—	—	—	—	—	—	—	—
**Interventions**											
	Valproate dosage	—	—	✓	✓	✓	—	—	—	—	—
**Assessments**											
	Clinical assessment	✓	✓	✓	✓	✓	✓	✓	✓	—	—
	CAM-ICU^c^, GCS^d^, and RASS^e^	—	—	✓	✓	✓	✓	✓	✓	—	—
	Etiological investigation	—	—	✓	✓	✓	✓	✓	✓	—	—
	Standard EEG^f^	—	—	—	—	—	✓	✓	✓	—	—
	SOFA^g^	—	—	✓	—	✓	✓	✓	✓	—	—
	SAPS-II^h^	—	—	—	—	—	✓	—	—	—	—
	FAB^i^, MMSE^j^, GOSE^k^, and SF-36^l^	—	—	—	—	—	—	—	—	—	✓
	Valproate adverse effects reporting	—	—	✓	—	✓	✓	✓	✓	—	—
	Primary outcome	—	—	—	—	—	—	—	✓	—	—
	Secondary outcome	—	—	✓	—	✓	—	—	—	—	—
	End of trial criteria	—	—	—	—	—	—	—	—	—	—

^a^✓: visit is scheduled.

^b^Absence of visits.

^c^CAM-ICU: Confusion Assessment Method for the Intensive Care Unit.

^d^GCS: Glasgow Coma Scale.

^e^RASS: Richmond Agitation-Sedation Scale.

^f^EEG: electroencephalogram.

^g^SOFA: sepsis-related organ failure assessment.

^h^SAPS-II: Simplified Acute Physiology Score.

^i^FAB: Frontal Assessment Battery.

^j^MMSE: Mini-Mental State Examination.

^k^GOSE: Glasgow Outcome Scale-Extended.

^l^SF-36: Short Form-36.

From the day of inclusion to day 7, a second 30-minute standard EEG was performed. From inclusion to day 15, patient monitoring was standardized to assess the evolution of GCSE, study drug side effects, and ICU-related complications (Table 1).

The primary end point was collected on day 15. ICU-related secondary end points were collected at the time of ICU discharge. From ICU to hospital discharge, the recurrence of seizures and changes in antiepileptic therapy were recorded. At the time of discharge, the patient was given a prescription for his antiepileptic treatment, its biological monitoring, and an appointment with a referral neurologist 3 months later. At day 90, vital, cognitive, and functional statuses were assessed by the referral neurologist or intensivist either by phone or through a medical examination (Table 1).

### Sample Size

The study was powered to detect an absolute increase of 20% in the rate of patients discharged alive at day 15 with a power of 90% and a two-sided 5% alpha risk, assuming this rate would be 50% in the control arm. Accordingly, the sample size was 124 patients per group. To account for the decrease in power owing to potential errors in the administration of the allocated treatment, this number was increased to 150 per arm. Therefore, the study initially planned to enroll a maximal sample size of 300 patients.

In fact, following the inclusion of the 245th patient, the RCT was discontinued owing to difficulties in recruiting. The decision was made because 98.8% (245/248) of the calculated sample size (n=248) had been enrolled and the associated decrease in power would be less than 1%. In addition, no treatment allocation error was noted, so there was no reason to target 300 patients.

### Recruitment

The study took place in 20 ICUs, which had been selected based on the interest expressed by local physicians, expertise in managing status epilepticus, their capacity to recruit eligible patients (ie, at least one patient per month), and RCT constraints as well as their easy access to a neurological department. A research assistant was available daily at every participating center to screen patients for inclusion. The steering committee met each month. A centralized phone and email center answered participating centers’ questions regarding patient eligibility or management and declaration of any adverse event during the trial period. A newsletter was sent monthly, informing the participating centers on the number of patients included, main study constraints, and any protocol modifications.

### Assignment of Interventions: Allocation

#### Sequence Generation

The randomization list was balanced between arms generated by the study statistician using permutation blocks of varying size (block of 2 or 4 patients, each with probability 0.5). Randomization was stratified by age group (≤65 or >65 years), center, and presence of *acute primary brain injury*. Stratification by age was performed, as it is a well-established prognostic factor for outcome of patients with GCSE.

#### Concealment Mechanism

Randomization and concealment were ensured using a web-based system accessible at each study center and managed by the clinical research unit, which had no role in patient recruitment.

#### Implementation

The allocation sequence was generated by the study statistician. Patient enrollment was ensured by the participating center investigator.

### Assignment of Interventions: Blinding

#### Who Will Be Blinded

The promoter provided the centers with sequentially numbered and sealed treatment boxes of identical appearance for either VPA or placebo. Boxes were prepared, coded, and shipped to participating sites by the Agence Générale des Equipements et Produits de Santé—Assistance Publique-des Hôpitaux de Paris (AP-HP, Paris France). These boxes contained all the elements needed to prepare the allocated treatment. The number of a given box related to the treatment unit number provided at the end of the randomization procedure. Reconstitution of the treatment was carried out (1) either in the pharmacy of the participating site, provided that reconstitution could be performed rapidly at any time of the day, or (2) in the ICU by an out-of-protocol nurse, who was not involved in patient management, monitoring, or follow-up. This procedure ensured a double-blind design, as the investigator and the rest of the ICU team remained unaware of treatment allocation.

The randomization sequence was concealed from patients, staff members, investigators, members of the independent Data Safety Monitoring Board (DSMB), and the sponsor.

#### Procedure for Unblinding if Needed

Unblinding was permissible but had to be explained by the investigator to either the antipoison center (Paris, France) or to the promoter.

### Data Collection and Management

#### Plans for Assessment and Collection of Outcomes

Baseline characteristics on admission were systematically collected by the center investigator: demographic and anthropometric data, location before ICU admission (community, hospital, or long-term facility); date and time of ICU admission, preexisting comorbidities using Knaus and McCabe scores, history of epilepsy and preexisting antiepileptic treatment, and other preexisting neurological diseases.

GCSE characteristics were recorded: circumstance of onset; focal or generalized onset; and time, type, and dosage of the AED administered. Neurological assessment included the GCS and occurrence of focal neurological deficit at any time. Paraclinical assessment of GCSE included the date and time of EEG, EEG features, brain imaging, and cerebrospinal fluid analysis when available. Finally, the etiology of GCSE was recorded and classified as acute, remote, progressive, or unknown [21].

Severity of critical illness was determined using the Simplified Acute Physiology Score II and Sequential Organ Failure Assessment score. Over the first 24 hours, vital signs including core temperature as well as hematologic and biochemical data, plasma level of creatine kinase, ammonia, and plasma level—human chorionic gonadotropin for women of childbearing age were recorded.

Patients were followed up for 90 days. From randomization to day 15, we recorded vital signs, Sequential Organ Failure Assessment score, need for mechanical ventilation or renal replacement therapy, and results of standard laboratory tests. GCSE assessment included date and time of cessation of clinical seizures, recurrence of seizure if any, evolution to refractory and superrefractory GCSE, as well as the date and results of EEG. The type and dose of the maintenance AED was also recorded. Neurological status was assessed daily by recording the GCS, Richmond Assessment Sedation Scale and Confusion Assessment Method in the ICU, type of sedation, and existence of focal neurological signs. At the time of ICU discharge, we collected information on the duration of sedation and mechanical ventilation, time for arousal, length of ICU stay, and antiepileptic treatment. At the time of hospital discharge, we collected the date of recurrence of seizure and GCSE, length of hospital stay, and destination (home, hospital, or long-term facility). In each participating center, the final diagnosis of GCSE and its cause were routinely confirmed by a neurologist. This evaluation will enable the identification of psychogenic GCSE, which can amount up to 10% of the cohort. No adjudicators were provided in our study to confirm the GCSE.

At day 90, we assessed the epileptic status by recording the recurrence of seizures or of GCSE, modification of antiepileptic therapy, vital status by collecting the date of death when appropriate, and the cognitive status by assessing the Extended Glasgow Outcome Scale, Mini-Mental State Examination, Frontal Assessment Battery, and quality of life using the Short Form-36. Day 90 assessment was performed by the referral neurologist or intensivist during the consultation or through a phone interview.

At the end of the trial, plasma VPA levels before, 15 minutes, and 12 hours after inclusion as well as the centralized interpretation of EEGs will be collected.

#### Plans to Promote Participant Retention and Complete Follow-Up

The participating teams were monthly informed of the course of the study and reminded of the main elements of the trial, notably concerning the follow-up.

#### Data Management

Data management and statistical analysis were performed independently of the sponsor and investigators by the clinical research unit (*Unité de Recherche Clinique, Hôpital Ambroise Paré, Boulogne, France*) and by the Center of Clinical Epidemiology (*Centre d’épidémiologie clinique, Hotel-Dieu, Paris, France*), respectively. Data entry occurred at enrolling sites by the investigator using a web-based data entry system.

An e-CRF (electronic Clinical Report Form) was developed by the clinical research unit using dedicated software (CleanWeb) to facilitate data control and monitoring. Each patient was assigned a unique ID that was used to index the e-CRF and related study documents.

All information required by the protocol had to be entered into the e-CRF. Data were recorded in the e-CRF as and when they were obtained. Any missing data had to be coded. In addition, the coherence of the entered data was immediately verified, because of inbuilt consistency checks.

Data monitoring was performed by the sponsor (*AP-HP; Délégation de la Recherche Clinique d’Ile de France, DRRC*). This project was classified as a C risk based on the AP-HP risk level classification, meaning that a high level of monitoring occurred, aimed at determining whether centers adhere to the protocol and the various circuits put in place to check the completeness of the e-CRF, to ensure patient safety (adverse events or serious adverse events), and follow-up in accordance with the applicable regulations. A clinical research associate (CRA) appointed by the sponsor is responsible for the good completion of the study and for collecting, documenting, recording, and reporting all handwritten data, in accordance with the Standard Operating Procedures applied within the Clinical Research and Innovation Department and in accordance with Good Clinical Practices as well as the statutory and regulatory requirements. During these visits, the following elements are reviewed:

- Written consent
- Safety and rights of subjects are being protected
- Compliance with the study protocol and with the procedures defined therein
- Quality of data collected in the CRF: accuracy, missing data, consistency of the data with the *source* documents (medical files, appointment books, original copies of laboratory results, etc). Data are authentic, accurate, and complete
- Management of the treatments used

Visits to pharmacies were also carried out in a way that verified compliance with the pharmaceutical circuit.

Baseline characteristics, eligibility criteria, primary outcome, and serious adverse events reported in the CRF were systematically checked against the original chart for all research participants by the CRA. In addition, for one-third of the study population, all data reported in the CRF were validated against the patient’s original chart. Serious adverse events and major protocol violations were reported to the DRRC, Agence Nationale de la Sécurité du Médicament (ANSM), and Comité de protection des personnes (CPP).

At the end of the study, after clarification of discrepancies (data cleaning) and data validation, the database was frozen and transmitted to the statistician following procedures established by the promoter.

Each patient participated in the trial for 90 days. Premature study withdrawal occurred on request of the patient or next of kin, and their reasons were recorded in the CRF and patient’s medical file. Withdrawn patients were not replaced. Conversely, patients who were lost to follow-up or did not receive the randomly assigned treatment were not considered to be prematurely withdrawn from the trial.

### Confidentiality

As for any clinical research supported by the AP-HP, processing of personal data complied with the methodological recommendations of the MR001 reference established by the French Data Protection Authority (*Commission Nationale de l’Informatique et des libertés*) in January 2006 for biomedical research. During and after the clinical research, all data collected concerning the participants and sent to the sponsor by the investigators (or any other specialized collaborators) are rendered nonidentifying. Under no circumstances shall the names and addresses of the participants involved be shown. Only the participant’s initials are recorded, accompanied by an encoded number specific to the study indicating the order of enrollment. Moreover, all nominal data were erased on the copies of the source files that were used for documentation of the research.

### Plans for Collection and Laboratory Evaluation and Storage of Biological Specimens for Genetic or Molecular Analysis in This Trial and Future Use

There were no genetic or molecular analyses planned.

### Statistical Methods

#### Statistical Methods for Primary and Secondary Outcomes

The comparison between arms will be adjusted for stratification variables (ie, age group and center) as recommended [22] as well as the presence of acute brain injury at inclusion, which is a major prognostic variable. The center will be considered as a random effect. In addition, two analyses will be performed according to age category (cutoff at 65 years) and acute brain injury.

Finally, unadjusted analyses will be performed for sensitivity analyses. Binary outcomes will be analyzed using logistic regression. Absolute risk reductions will be obtained using a binomial model with an identity link [23]. For time-to-event outcomes, Kaplan-Meier survival curves or cumulative incidence curves will be estimated, and the treatment effect will be analyzed using Cox proportional hazards regression. For continuous outcomes, mixed linear regression will be used, possibly after variance stabilizing transformation.

All tests will be two-sided, at a 0.05 significance level.

#### Interim Analyses

We neither planned nor performed an interim analysis.

#### Methods for Additional Analyses (eg, Subgroup Analyses)

Adherence to national guidelines on anticonvulsant therapy, control of secondary brain insult, etiological investigations, and neurological monitoring was strongly recommended to minimize heterogeneity in GCSE management. Moreover, randomization was stratified by center to limit any center effect. Finally, both randomization and statistical adjustments are likely to minimize discrepancies between therapeutic groups.

#### Methods in Analysis to Handle Protocol Nonadherence and Any Statistical Methods to Handle Missing Data

Statistical analysis will be performed according to the intent-to-treat principle, after all patients have completed the 90-day follow-up. Accordingly, all patients will be analyzed in the arm they were allocated to, regardless of the protocol deviations. In addition, missing outcome data will be imputed. Before data analysis, a detailed statistical analysis plan will be issued by the study statistician. A comprehensive report of the statistical analysis will be issued, following the Consolidated Standards of Reporting Trials (CONSORT) statement recommendations. Any change in the analysis plan will be justified in this final report.

Although no missing data are expected for the primary outcome, the maximum bias method will be used for the analysis of the primary outcome, replacing missing data with a success in the control arm and by a failure in the experimental arm. For secondary outcomes, missing data will be handled by multiple imputations by chained equations. A sensitivity analysis will be performed by analyzing only complete cases.

#### Plans to Give Access to the Full Protocol, Participant-Level Data, and Statistical Code

Persons with direct access in accordance with the laws and regulations in force, in particular, articles L.1121-3 and R.5121-13 of the public health code (eg, investigators, persons responsible for quality control, monitors, clinical research assistants, auditors, and others involved in collaborating on trials), take all necessary precautions to ensure the confidentiality of information relating to the tested drugs, the trial, the persons involved, especially with regard to their identity and the results obtained. The data collected by these people during quality controls or audits are then made anonymous.

### Oversight and Monitoring

#### Composition of the Coordinating Centre and Trial Steering Committee

The steering committee includes Dr Hervé Outin, Dr Bernard Clair, and Professor Tarek Sharshar, who were the initiators of the project. The steering committee, with the biostatistician and the promoter’s representatives (Direction de la recherché Clinique et du développement—DRCD-headquarters and DRCD—Unité de Recherche Clinique) appointed for this research, may decide during the trial the procedures to be followed, taking note of the recommendations of the independent supervisory committee. They will define the general organization and conduct of the research and coordinate the information. The steering committee has decided the methodology and will decide during the course of the trial the conduct to be followed in case of unforeseen matters and will monitor the progress of the research, particularly in terms of tolerance and adverse events.

#### Composition of the Data Monitoring Committee, Its Role, and Reporting Structure

The DSMB was established by the sponsor. Its primary mission was to monitor safety data. It was composed of experts in Critical Care Medicine, Neurology and Statistics, who were not involved in the trial but had full access to the raw data. The DSMB was composed of Dr Nicolas Melé (Neurology-Sainte-Anne Teaching Hospital, Paris), Dr Olivier Lesieur (Intensive Care Medicine- General Hospital—La Rochelle), and Dr Cédric Laouenan (biostatistics Bichat Teaching Hospital, Paris). The DSMB was operated in accordance with the sponsor’s procedures. The DSMB worked in an advisory capacity only, and the sponsor retained all decision-making authority. This committee met once a year.

#### Adverse Event Reporting and Harms

A centralized phone and email center answered participating centers questions regarding patient eligibility or management and declaration of any adverse event during the trial period. A newsletter was sent monthly, informing participating centers on the number of patients included, main study constraints, and any protocol modifications.

Baseline characteristics, eligibility criteria, primary outcome, and serious adverse events reported in the CRF were systematically checked against the original chart for all research participants. In addition, for one-third of the study population, all data reported in the CRF were validated against the patient’s original chart. Serious adverse events and major protocol violations were reported for DRRC, ANSM, and CPP.

#### Frequency and Plans for Auditing Trial Conduct

All data, documents, and reports may be subject to regulatory audits and inspections. These audits and inspections cannot be refused on the grounds of medical secrecy.

An audit can be carried out at any time by individuals appointed by the sponsor and independent of those responsible for the research. The aim of the audits is to ensure the quality of the study, the validity of the results, and compliance with the legislation and regulations in force.

The individuals in charge of managing and monitoring the study agreed to comply with the sponsor’s requirements and with the competent authority regarding study audits or inspections.

An audit may encompass all stages of the study, from the development of the protocol to the publication of the results, including the storage of the data used or produced as part of the study. For this study, we did not conduct an audit in any of the participating centers.

#### Plans for Communicating Important Protocol Amendments to Relevant Parties (eg, Trial Participants, Ethical Committees)

All substantial modifications to the protocol by the coordinating investigator were sent to the sponsor for approval. After approval, the sponsor obtained approval from the CPP (Research Ethics Committee) and authorization from the ANSM within the scope of their respective authorities before the amendment can be implemented.

The information note and the consent form have been revised, particularly in the case of a substantial amendment to the study.

#### Dissemination Plans

Neither the study sponsor nor the study funder had any role in designing the trial; managing, analyzing, or interpreting the data; writing the report; or deciding to submit the report for publication.

#### Patient and Public Involvement

No patient involved.

### Availability of Data and Materials

In accordance with Good Clinical Practices (1) the sponsor is responsible for ensuring all parties involved in the study agree to guarantee direct access to all locations where the study will be carried out, the source data, the source documents, and the reports, for the purposes of the sponsor’s quality control and audit procedures or inspections by the competent authority; and (2) the investigators allow individuals in charge of monitoring quality control to have access to the documents and personal data strictly necessary for these tasks, in accordance with the statutory and regulatory provisions in force (Articles L.1121-3 and R.5121-13 of the French Public Health Code).

The AP-HP had full access to patients’ charts and checked all data recorded in the electronic CRF against the original charts. All information required by the protocol had to be provided in the electronic logbook and an explanation provided by the investigator for each missing data.

### Ethics Approval and Consent to Participate

The protocol was approved by all investigators on January 25, 2012. The scientific and financial aspects were independently approved by the national jury of the Clinical Research Hospital Program in 2010, and the Ministry of Health confirmed funding under contract number AOM10268. The protocol and qualification of all investigators were approved by the Ethics Committee (CPP) of Saint-Germain-en-Laye, France, on May 14, 2012. CPP allowed for the waiver of consent and deferred consent. The trial was registered at ClinicalTrials.gov (identifier NCT01791868; registered on May 2012).

Written informed consent had to be obtained from all participants. Written informed consent of the patient was obtained by the investigator of the participating center. In case of impaired consciousness, the investigator sought written consent from the next of kin. If the latter was not present, the patient could be included as deferred consent was approved by the Ethics Committee, according to the French law (Article L1122-1-2 du Code de la Santé Publique). As soon as the patient’s status allowed, written informed consent for the continuation of the research and analyses of the data was obtained. A copy of the consent form was provided to every patient. The investigator had to keep the original copy in his archives for a minimum of 15 years. A third copy was archived by the promoter. Patients or the public were not involved in the design, conduct, reporting, or dissemination plans of our research.

## Results

### Inclusion Status

The first patient was recruited on February 18, 2013, and the last patient on July 7, 2018. The study was never suspended. The study sponsor, steering committee, investigators, pharmacists, and study statisticians remained blinded to study treatments throughout the trial. Data management is ongoing. Release of the results is planned for the end of 2021.

### Amendments

There were 10 amendments to the study protocol (Table 2). All amendments were approved by the investigators, the study statistician, AP-HP, CPP, and ANSM.

**Table 2:** Study amendments.

Amendment number	Description	Date (CPP^a^-ANSM^b^)
1	Withdrawal of center 13 Bordeaux Modification of exclusion criteria Addition of sodium valproate derivatives Precision on the Prothrombin time and Factor V assay algorithm Suppression of the ATICE^c^ score Changes made to the balance sheets: Adding a balance sheet module before inclusion Suppression of SAPS-II^d^ at H0 at inclusion Modification of bilirubin at inclusion, in the first 24 hours, from the 2nd to the 15th day of inclusion Modification of the SAPS-II in the first 24 hours CPK^e^ change in the first 24 hours Add GCS^f^, RASS^g^ score and CAM-ICU^h^ score to the resuscitation output Numbering changes Amended Protocol v2.0 of 20/10/2012	ANSM: 11/27/2012; CPP: 11/12/2012
2	Modification of the principal investigators: Center 006 Beaujon, Dr Catherine Paugam-Burtz Center 009 Strasbourg, Dr Marie-Line Harlay Center 015 Pontoise, Dr Pascal Blanc	CPP: 05/12/2013
3	Possible randomization of patients even if the biological results were not obtained within the deadlines Amended Protocol v3.0 of 24/06/2013	ANSM: 07/29/2013; CPP: 09/16/2013
4	Modification of the inclusion criteria: Admission to resuscitation for GCSE^i^, that is, persistent or recurrent generalized convulsions without regaining consciousness for more than 5 minutes, and antiepileptic management <6 hours (if the GCSE is controlled at the time of inclusion) or <24 hours (if the GCSE has persisted or recurs) Age≥18 years Deletion of the SAPS-II at H12 calculation Dosage of depakinemia at T0, T15 minutes, and T12 hours 15 minutes. The sampling and shipping procedures are being finalized; we will come back to each center to discuss how to put them into practice Addition of two centers (Reunion Island and Montpellier) Amended Protocol v4.0 of 26/02/2014	ANSM: 05/02/2014; CPP: 07/01/2014
5	Modification of the inclusion criterion on admission to intensive care Modification of the criteria for noninclusion: Forms of states of epilepsy Liver test The prior taking of VPA Sampling procedure Amended Protocol v5.0 of 04/12/2014	ANSM: 02/03/2015; CPP: 02/13/2014
6	Changes to the emergency and prosecution ICFs (version 2) Changes to the criteria for noninclusion No. 5: pregnancy, especially eclampsia - check by a systematic pregnancy test No. 11: patient under guardianship No. 12: patient who has already been included in this protocol and who has completed the clinical trial During the 3-month checkup, it will be asked if a pregnancy was initiated between the inclusion and the visit at 3 months, and if so, the date of the beginning of the pregnancy will be collected Amended Protocol v6.0 of 01/02/2016 ICF v2.0 of 01/02/2016	ANSM: 03/18/2016; CPP: 05/24/2016
7	Modification of the principal investigator of the Lariboisière center, Pr Bruno Megarbane Modification of blood sampling roadmaps Amended Protocol v7.0 of 22/06/2016	CPP: 11/04/2016
8	Extension of the 12-month inclusion period Amended Protocol v8.0 of 02/01/2017	CPP: 04/29/2017
9	Extension of the inclusion period by 6 months Amended Protocol v9.0 of 19/02/2018	ANSM: 03/26/2018; CPP: 05/07/2018
10	The addition of an exclusion criterion (patients of childbearing age between 18 and 50 years), following an ANSM alert	ANSM: 07/19/2018; CPP: 10/18/2018

^a^CPP: Comité de protection des personnes (institutional review board).

^b^ANSM: Agence Nationale de Sécurité du Medicament (French National Agency for Drugs Safety).

^c^ATICE: adaptation to the intensive care environment.

^d^SAPS-II: Simplified Acute Physiology Score II.

^e^CPK: creatine phosphokinase.

^f^GCS: Glasgow Coma Scale.

^g^RASS: Richmond Agitation-Sedation Scale.

^h^CAM-ICU: Confusion Assessment Method for the Intensive Care Unit.

^i^GCSE: generalized convulsive status epilepticus.

### Study Follow-Up

The DSMB met five times. The DRRC organized data monitoring and quality audits. Baseline characteristics, eligibility criteria, primary outcome, and serious adverse events reported in the CRF were systematically checked against the original chart for all research participants. In addition, for one-third of the study population, all data reported in the CRF were validated against the patient’s original chart. Serious adverse events and major protocol violations were reported for DRRC, ANSM, and CPP. The study coordinator had quarterly face-to-face meetings with the DRRC, AP-HP, and independent pharmacists to monitor trial conduct according to the highest standard for protection of research participants. All randomized patients completed follow-up for the primary outcome and 180-day mortality data.

## Discussion

### Novelty of the Study

This multicenter, parallel-group, double-blind RCT was designed to determine whether VPA improves the outcome of patients admitted to the ICU for GCSE as an adjuvant therapy to recommended first- and second-line AEDs. This hypothesis was based on the antiepileptic and potential neuroprotective properties of VPA, which could improve seizure control and minimize GCSE-related additional brain injury. The amendments made to the protocol were aimed at improving patient recruitment.

One may argue that another AED could have been proposed instead of VPA. We opted for VPA mainly because it was not recommended by the national guidelines at the time of study design as second-line AED for GCSE, enabling us to avoid the risk of overdose and to undertake a stepwise strategy. In addition, we did not choose to assess levetiracetam, as it was being tested as adjuvant therapy to the first-line AED [5]. Adjuvant levetiracetam was not beneficial. Moreover, VPA is well tolerated and is not contraindicated with recommended second-line and most maintenance AEDs. The ESETT trial does not undermine the relevance of our RCT, as it showed that VPA is as efficient as levetiracetam and fosphenytoin as a second-line AED [16]. Therefore, it is likely that VPA will not be the second-line AED administered in many patients with GCSE, who could then be treated with VPA, if our RCT shows a benefit of VPA. Finally, if our RCT is positive, it would be necessary to test another AED as adjuvant treatment.

### Randomization Procedure

Selection biases were minimized and homogeneity between the two groups was ensured by the double-blind design. First, the random list for allocating interventions was computer-generated by an independent statistician. Randomization was centralized through a secured website using permutation blocks, the size of which was unknown to research participants. Second, a centralized procedure for masking VPA and placebo was used; pharmacists received sealed boxes containing either treatment in identical forms. Reconstitution of the treatment was done by an *out-of-protocol* pharmacist or nurse. Therefore, research participants were unable to anticipate or identify patients’ allocation. Third, hospital staff, investigators, pharmacists, and outcome assessors remained blinded for short- and long-term outcomes until public release of trial findings, to prevent any detection biases. Finally, there were no obvious attrition biases. No patient was lost to follow-up for the primary end point. Although reporting the study design and statistical analysis plan after completion of patient recruitment might be a potential source of bias, this was necessary to detail the way the trial was conducted and amended. We neither planned nor performed an interim analysis.

### End Points

The primary end point (ie, discharge from hospital at day 15) might not be sufficiently specific but also liable to various biases. Indeed, hospital discharge depends on factors related to the patient’s social and economic condition, as well as on hospital organization and health care facilities. However, randomization theoretically limits the risk of differential bias between the VPA and placebo groups. Therefore, we assumed that hospital status at day 15 would reflect the control of the epileptic process, its neurotoxic consequences, and underlying cause. Finally, an improvement in hospital status at day 15 is medically, socially, and economically relevant. A recent French clinical trial in a comparable population found length of hospital stay of about 19 days [9]. Although the median length of hospital stay was 3 days in a recent trial comparing VPA to levetiracetam and fosphenytoin, the included population is not comparable to our cohort in terms of age, course, and severity of GCSE [16]. Indeed, the included patients were 2 years old or more, and only half of the included patients were admitted to the ICU [16].

Our secondary end points are conventional, including information on the GCSE course such as duration, progress to refractory GCSE, EEG characteristics, and long-term control of epilepsy. We acknowledge that the duration of seizure would have been better assessed using a continuous EEG;, however, this was not available in most participating centers. It is likely that the clinical assessment at 3 months will be missing for a large number of patients. Indeed, in recent trials, less than 30% of the included patients were assessed at 3 months for epileptic and cognitive status, indicating the difficulty of follow-up of these patients [9].

### GCSE

One may argue that the studied population could be heterogeneous in terms of severity, underlying cause, and pre-ICU management of GCSE. Indeed, both mechanically ventilated and nonmechanically ventilated patients were included, despite the fact that the need for mechanical ventilation mainly reflects the depth of consciousness impairment, likely to be related to the early severity of GCSE or its etiology. As age and acute brain injury are the two main prognostic factors in GCSE, we planned to adjust statistical analysis on these demographic and etiological predictors. Adherence to national guidelines on anticonvulsant therapy, control of secondary brain insult, etiological investigations, and neurological monitoring was strongly recommended to minimize heterogeneity in GCSE management. Moreover, randomization was stratified by center to limit any center effect. Finally, both randomization and statistical adjustments are likely to minimize discrepancies between therapeutic groups.

Therefore, the VALSE multicenter RCT is appropriately designed to address an original issue: the role of VPA as an adjuvant neuroprotective therapy in GCSE. VALSE aims to include a representative population of patients admitted to the ICU for GCSE, who will go on to receive standardized GCSE management. The objective of obtaining a 20% increase in the rate of patients with GCSE discharged alive from hospital at day 15 is clinically relevant and is also easily achievable and assessable. The trial is designed to integrate adjustments on the main outcome predictors and to collect potential confounding factors. Therefore, VALSE will provide reliable and relevant data that might improve ICU management of GCSE. At present, data analysis is still pending, and all parties involved in the trial remain blinded.

### Strengths and Limitations of the Study Summary

This is the first multicenter randomized double-blind controlled trial that assesses whether VPA can be useful as an adjuvant therapy to recommend first- and second-line AED to improve the outcome of GCSE. This RCT has been designed and powered to address this major issue, as GCSE is still associated with high mortality and morbidity. The trial is based on a clinically relevant primary end point, that is, hospital status at day 15, as it reflects the control of the epileptic process, its neurotoxic consequences, and underlying cause. This trial concerns only adult patients admitted to the ICU for GCSE.

## Appendix

Multimedia Appendix 1 CONSORT Checklist (2010).

## Abbreviations

AED: antiepileptic drug
ANSM: Agence Nationale de la Sécurité du Médicament (National Agency for Drugs Safety)
AP-HP: Assistance Publique-Hôpitaux de Paris
CPP: Comité de protection des personnes (Institutional Review Board)
CRA: clinical research associate
DRCD: Direction de la recherché Clinique et du développement
DRRC: Direction Régionale de la Recherche Clinique (Regional Clinical Research Agency)
DSMB: Data Safety Monitoring Board
EEG: electroencephalogram
ESETT: Established Status Epilepticus Treatment Trial
GCS: Glasgow Coma Scale
GCSE: generalized convulsive status epilepticus
ICU: intensive care unit
RCT: randomized clinical trial
VALSE: valproic acid as an adjuvant treatment for generalized convulsive status epilepticus
VPA: valproic acid

## References

[1] Eriksson K, Metsäranta P, Huhtala H, Auvinen A, Kuusela A-, Koivikko M (2005). Treatment delay and the risk of prolonged status epilepticus. Neurology.

[2] Sculier C, Gaínza-Lein M, Sánchez Fernández I, Loddenkemper T (2018). Long-term outcomes of status epilepticus: a critical assessment. Epilepsia.

[3] Glauser T, Shinnar S, Gloss D, Alldredge B, Arya R, Bainbridge J, Bare M, Bleck T, Dodson WE, Garrity L, Jagoda A, Lowenstein D, Pellock J, Riviello J, Sloan E, Treiman DM (2016). Evidence-based guideline: treatment of convulsive status epilepticus in children and adults: report of the guideline committee of the American epilepsy society. Epilepsy Curr.

[4] Trinka E, Cock H, Hesdorffer D, Rossetti AO, Scheffer IE, Shinnar S, Shorvon S, Lowenstein DH (2015). A definition and classification of status epilepticus-report of the ILAE task force on classification of status epilepticus. Epilepsia.

[5] Navarro V, Dagron C, Elie C, Lamhaut L, Demeret S, Urien S, An K, Bolgert F, Tréluyer J, Baulac M, Carli P (2016). Prehospital treatment with levetiracetam plus clonazepam or placebo plus clonazepam in status epilepticus (SAMUKeppra): a randomised, double-blind, phase 3 trial. Lancet Neurol.

[6] Leppik IE (1983). Double-blind study of lorazepam and diazepam in status epilepticus. J Am Med Assoc.

[7] Silbergleit R, Durkalski V, Lowenstein D, Conwit R, Pancioli A, Palesch Y, Barsan W (2012). Intramuscular versus intravenous therapy for prehospital status epilepticus. N Engl J Med.

[8] Treiman DM, Meyers PD, Walton NY, Collins JF, Colling C, Rowan AJ, Handforth A, Faught E, Calabrese VP, Uthman BM, Ramsay RE, Mamdani MB (1998). A comparison of four treatments for generalized convulsive status epilepticus. Veterans affairs status epilepticus cooperative study group. N Engl J Med.

[9] Legriel S, Lemiale V, Schenck M, Chelly J, Laurent V, Daviaud F, Srairi M, Hamdi A, Geri G, Rossignol T, Hilly-Ginoux J, Boisramé-Helms J, Louart B, Malissin I, Mongardon N, Planquette B, Thirion M, Merceron S, Canet E, Pico F, Tran-Dinh Y, Bedos J, Azoulay E, Resche-Rigon M, Cariou A (2016). Hypothermia for Neuroprotection in Convulsive Status Epilepticus. N Engl J Med.

[10] Brandt C, Gastens AM, Sun MZ, Hausknecht M, Löscher W (2006). Treatment with valproate after status epilepticus: effect on neuronal damage, epileptogenesis, and behavioral alterations in rats. Neuropharmacology.

[11] Outin H (2009). Emergency and intensive care unit management of status epilepticus Société de réanimation de langue française Experts Recommendations: the transient triumph of the followers of Sisyphus. Rev Neurol (Paris).

[12] Trinka E, Kälviäinen R (2017). 25 years of advances in the definition, classification and treatment of status epilepticus. Seizure.

[13] Brigo F, Bragazzi N, Nardone R, Trinka E (2016). Direct and indirect comparison meta-analysis of levetiracetam versus phenytoin or valproate for convulsive status epilepticus. Epilepsy Behav.

[14] Misra UK, Kalita J, Patel R (2006). Sodium valproate vs phenytoin in status epilepticus: a pilot study. Neurology.

[15] Gilad R, Izkovitz N, Dabby R, Rapoport A, Sadeh M, Weller B, Lampl Y (2008). Treatment of status epilepticus and acute repetitive seizures with i.v. valproic acid vs phenytoin. Acta Neurol Scand.

[16] Kapur J, Elm J, Chamberlain JM, Barsan W, Cloyd J, Lowenstein D, Shinnar S, Conwit R, Meinzer C, Cock H, Fountain N, Connor JT, Silbergleit R (2019). Randomized trial of three anticonvulsant medications for status epilepticus. N Engl J Med.

[17] Riviello JJ, Claassen J, la Roche SM, Sperling MR, Alldredge B, Bleck TP, Glauser T, Shutter L, Treiman DM, Vespa PM, Bell R, Brophy GM, Neurocritical Care Society Status Epilepticus Guideline Writing Committee (2013). Treatment of status epilepticus: an international survey of experts. Neurocrit Care.

[18] Aranda A, Foucart G, Ducassé JL, Grolleau S, McGonigal A, Valton L (2010). Generalized convulsive status epilepticus management in adults: a cohort study with evaluation of professional practice. Epilepsia.

[19] Nanau RM, Neuman MG (2013). Adverse drug reactions induced by valproic acid. Clin Biochem.

[20] Ferlisi M, Shorvon S (2012). The outcome of therapies in refractory and super-refractory convulsive status epilepticus and recommendations for therapy. Brain.

[21] Berg A, Berkovic S, Brodie M, Buchhalter J, Cross J, van Emde WB, Engel J, French J, Glauser TA, Mathern GW, Moshé SL, Nordli D, Plouin P, Scheffer IE (2010). Revised terminology and concepts for organization of seizures and epilepsies: report of the ILAE commission on classification and terminology, 2005-2009. Epilepsia.

[22] Lewis JA (1999). Statistical principles for clinical trials (ICH E9): an introductory note on an international guideline. Statist. Med.

[23] Pedroza C, Truong VT (2016). Performance of models for estimating absolute risk difference in multicenter trials with binary outcome. BMC Med Res Methodol.

